# Drug exposure: still relevant after all these years

**DOI:** 10.18632/oncotarget.27899

**Published:** 2021-04-13

**Authors:** David E. Gerber, William C. Putnam

**Keywords:** pharmacokinetics, pharmacodynamics, biomarkers, lung cancer

In a recent window-of-opportunity study, we repurposed an established antifungal agent as a potential cancer therapy [1]. Specifically, we administered itraconazole for 10–14 days to patients with early-stage, non-small cell lung cancer immediately prior to surgical resection. Such a trial design permits numerous opportunities to straightforwardly study the biologic effects of treatment, including serial blood collections, serial imaging studies, and pre- and post-tissue analysis. In this study, we analyzed circulating cytokines and angiogenic factors, tumor microvessel density and perfusion, developmental signaling pathways, and systemic and intra-tumoral drug concentrations.

Itraconazole, approved for invasive fungal infections such as aspergillosis, histoplasmosis, and candidiasis in the 1990s, has known antiangiogenic properties, and inhibits the Hedgehog developmental pathway [2, 3]. This latter characteristic is particularly important in Hedgehog-driven tumors such as basal cell carcinoma, for which the Hedgehog inhibitors vismodegib and sonidegib are FDA approved. Additionally, the Hedgehog pathway may have a role in other malignancies such as prostate cancer, gastrointestinal cancers, and medulloblastoma [4, 5]. Directly relevant to our current study, orally administered itraconazole is also known to have unpredictable and variable pharmacokinetic parameters, in part due to food and gastric pH effects on bioavailability [6] and effects of CYP3A4 and P glycoprotein [7, 8]. Because early studies of itraconazole demonstrated a clinically meaningful dose exposure-response relationship, monitoring itraconazole levels for both prophylactic and therapeutic indications has been recommended [9–11].

Given the known pharmacokinetic profile of itraconazole, it may not be entirely surprising that we observed a six-fold variation in itraconazole concentrations among the 13 subjects. This high level of variability could not be attributed to duration of therapy, concomitant medications, food intake, patient size, or kidney or liver function. Furthermore, our analyses demonstrated a profound and significant association between intraconazole concentrations (both plasma and intra-tumoral) and numerous efficacy measures, including reduction in tumor vasculature and tumor size.

Performing accurate and precise pharmacokinetic analyses in plasma, not to mention complex biologic matrices such as resected tumor tissue, requires careful planning and execution. This starts with careful coordination with well-trained clinical personnel to ensure that dosing and sample collection are carefully conducted and appropriately recorded. Although it may not ultimately matter if a blood draw is 10 minutes early or late, failing to record the actual collection time reliably may cause variability in PK determinations. Following collection, samples need to be processed according to a well-researched and validated procedure. During analytical method development and validation, sample processing variables including anticoagulant, storage conditions prior to processing, processing parameters such as centrifuge speed and time can all impact the ultimate results of the study; therefore, the impact of changes to those parameters (robustness) should be examined, established, and provided in specific guidance for clinical staff. For our study, we developed a written laboratory manual detailing dosing and sample collection, collection form templates, and pre-labeled vials to prevent sample mis-collection/identification.

Following collection, samples should be analyzed using an appropriately validated bioanalytical method. The FDA’s Guidance for Industry “Bioanalytical Method Validation” (May 2018) provides a solid framework for method development, validation, and sample analysis for most studies. In the current study, two liquid chromatography tandem mass spectrometric methods (LC-MS/MS) were developed and fully individually validated per FDA guidance for quantitative determination of itraconazole and hydroxyitraconazole in: (1) human plasma [method 1] and (2) human tissue [method 2]. This validated method was used to determine plasma and intra-tumoral concentrations of itraconazole and hydroxyitraconazole, which provided the data for pharmacokinetic analysis. Ultimately, use of this approach allowed for robust data collection regarding the kinetics of itraconazole at the individual patient level, thereby affording the ability to correlate the concentrations of itraconazole with markers of its efficacy at the individual patient level.

Perhaps in part due to these complexities, concentration-based treatment titration is rarely incorporated into clinical research or routine clinical care. Other published trials of itraconazole as a repurposed anti-cancer agent have not assessed drug levels to guide therapy administration [12, 13]. Conventional therapies needing regular evaluation of pharmacokinetic or pharmacodynamic parameters—such as the anticonvulsant phenytoin, the antiarrhythmic digoxin, and the anticoagulant warfarin—have been replaced by newer drugs without such requirements. At the same time, the field of oncology has experienced a major shift away from individualized dosing based on weight or body surface area (e.g., most conventional cytotoxic chemotherapeutic agents) toward fixed-dose agents such as biologics and molecularly targeted therapies.

Recent years have seen a veritable explosion in approved and experimental biomarkers to predict the efficacy of cancer therapies. These range from tumor histology (e.g., superior outcomes from the antimetabolite pemetrexed in squamous non-small cell lung cancer), to immunohistochemical features (e.g., hormone receptor expression for aromatase inhibitors or estrogen receptor modulators in breast cancer), to genomic profiles (e.g., *TRK* alterations for TRK inhibitors). With the advent of immune checkpoint inhibitors, exploratory biomarkers now reflect patient as well as tumor biology, such as gut microbiome or HLA type [14, 15]. Amid these promising and complex developments, let us not forget basic and essential questions such as drug dosing and exposure.
